# MRI-Based Mapping of Cerebral Propagation in Amyotrophic Lateral Sclerosis

**DOI:** 10.3389/fnins.2018.00655

**Published:** 2018-09-26

**Authors:** Hans-Peter Müller, Jan Kassubek

**Affiliations:** Department of Neurology, University of Ulm, Ulm, Germany

**Keywords:** amyotrophic lateral sclerosis, motor neuron disease, diffusion tensor imaging, fractional anisotropy, magnetic resonance imaging

## Abstract

Neuropathological studies revealed the propagation of amyotrophic lateral sclerosis (ALS) in a sequence of four separate disease-related regional patterns. Diffusion tensor imaging (DTI)-based analysis was established for the individual mapping of sequential disease spreading in ALS as the *in vivo* transfer to neuroimaging. The aim of this review is to summarize cross-sectional and longitudinal results of these technical approaches in ALS as an *in vivo* tool to image ALS propagation stages. This concept was also applied to restricted phenotypes of ALS, e.g., lower motor neuron disease (LMND) or primary lateral sclerosis (PLS). In summary, the regional disease patterns in the course of ALS have been successfully mapped by DTI *in vivo* both cross-sectionally and longitudinally so that this technique might have the potential as a read-out in clinical trials.

## Introduction

The potential of neuroimaging as a technical biological marker for cerebral microstructural alterations in neurodegenerative diseases like motor neuron disorders (MND) is under investigation (Turner et al., 2011, 2012). This review was designed to summarize diffusion tensor imaging (DTI)-based approaches for mapping the established propagation patterns in the brain in amyotrophic lateral sclerosis (ALS) and its variants (restricted phenotypes Ludolph et al., 2015). Classification of MND is a challenge of growing importance given that the therapeutic portfolio for ALS might expand in the future, as reflected in the efforts to revise the diagnostic criteria (Ludolph et al., 2015). With respect to the clinical presentation of ALS, the current revision of the El Escorial criteria addressed a validated staging system, and it was held that the development of non-invasive investigations including MRI will assist (Ludolph et al., 2015). For the staging concept, post-mortem studies of the brain pathology of ALS based on phosphorylated 43 kDa TAR DNA-binding protein (pTDP-43) revealed a possible dissemination in a regional sequence of four disease-related patterns (Braak et al., 2013; Brettschneider et al., 2013; Jucker and Walker, 2013), with the sequential protein pathology spreading initially from the motor neocortex toward the spinal cord and brainstem, followed by spreading to frontal, parietal and, ultimately, anteromedial temporal lobes (Ludolph and Brettschneider, 2015). This corticoefferent spreading model has been transferred *in vivo* to MRI-based concepts by *in silico* models (Schmidt et al., 2016), microstructural data (Kassubek et al., 2014, 2018b), and functional connectivity analysis (Schulthess et al., 2016). Specifically, DTI can be used to detect pathology within the corresponding neuronal white matter (WM) tracts and to obtain *in vivo* staging at an individual patient level by fiber-tract of interest (TOI)-based DTI mapping, i.e., a hypothesis-driven approach that revealed sequential involvement of the corresponding WM tracts in cross-sectional data (Kassubek et al., 2014) and longitudinal data (Kassubek et al., 2018b). To assess the axonal damage and myelin degradation, the statistical analysis of DTI metrics can be performed by various approaches: (1) unbiased voxelwise comparison by whole brain-based spatial statistics (WBSS) (Müller et al., 2012) or tractwise comparison by tract-based spatial statistics (TBSS) (Smith et al., 2006), or (2) hypothesis-guided tract-based quantification by analyzing DTI metrics in tract systems by probabilistic tools (Sarica et al., 2017), or TOI-based tractwise fractional anisotropy statistics (TFAS) (Müller et al., 2007b). In this review, results of DTI-based cross-sectional and longitudinal analyses in ALS were summarized including applications to clinical variants, i.e., lower motor neuron disease (LMND) and primary lateral sclerosis (PLS).

## Dti Data Analysis Techniques

The post-processing and statistical analysis of WBSS and TFAS was performed by use of the analysis software tensor imaging and fiber tracking (TIFT) (Müller et al., 2007a). In order to assess the axonal damage and myelin degradation, DTI metrics effects at the group level are reported by voxelwise WBSS comparison (Müller et al., 2012) and tract-based quantification by TOI-based TFAS (Müller et al., 2007b). Standard pre-processing procedures contain quality control of the DTI data including elimination of corrupted DTI volumes (Müller et al., 2011), motion correction of individual DTI data sets, in case of longitudinal data an alignment of baseline data and follow-up data by a halfway rigid-brain co-registration (Menke et al., 2014), normalization to the Montreal Neurological Institute (MNI) stereotaxic standard space (i.e., non-linear and iterative normalization to a study specific template – Müller et al., 2012), and, in case of DTI data from different scanners, a 3-D inter-protocol correction which can be applied ex post facto (Rosskopf et al., 2015). The covariate age should be regressed out due to an age dependency of FA values (Lim et al., 2015). In case of longitudinal analyses, the FA differences between the baseline and follow-ups were normalized to an identical time interval representing comparable disease durations for all patients before group level comparison as previously described in detail (Kassubek et al., 2018b), in order to control for variable follow-up intervals. Post-processing and statistical analysis was performed by a differentiated analysis, i.e., unbiased WBSS (Müller et al., 2012) that statistically compares voxelwise FA values of two subject groups and hypothesis-based tractwise quantification by analyzing FA values along tract systems (TFAS – Müller et al., 2007b).

Fiber tracts were reconstructed from an averaged DTI data set of MNI transformed controls’ data (Müller et al., 2007b) by a seed-to-target approach (Kassubek et al., 2014, 2018b); here, for a given pathway, the corresponding TOI is defined by all tracts that originate in a defined seed ROI and end in a target ROI. For quantification of the directionality of the underlying tract structures, the TFAS technique (Müller et al., 2007b) was applied. The four-stage corticoefferent sequential axonal spread of pTDP-43 has been transferred *in vivo* by a hypothesis-driven TOI-based analysis that revealed sequential involvement of the corresponding WM tracts in cross-sectional data (Kassubek et al., 2014) and longitudinal data (Kassubek et al., 2018b). Staging categorization for a given patient at the individual level is possible using an FA-based categorization scheme with sequential involvement of the specific tract structures (Kassubek et al., 2014, 2018b).

## *In Vivo* Transfer of the Staging Concept

### The TOI-Based Staging Approach

The hypothesis-guided TOI-based staging approach was suggested to image the neuropathologically proposed sequential progression of ALS in the respective cerebral tract systems, i.e., the CST (as a correlate of ALS-stage 1), the corticorubral and corticopontine tracts (ALS stage 2), the corticostriatal pathway (ALS stage 3), and the proximal portion of the perforant path (ALS stage 4) (Kassubek et al., 2014). The statistical analyses of TOIs showed differences between ALS patients and healthy controls for all tract systems; the significance level of the cross-sectional comparison at the group level in the corresponding fiber tracts was lower, the higher ALS-stage was (Kassubek et al., 2014). After a cross-sectional study with 111 ALS patients and 74 healthy controls with MRI data from 1.5T as well as at 3.0T scanners, a follow-up (mono-centre) study confirmed the results in 382 ALS patients and 149 healthy controls (Kassubek et al., 2018b). In a subsample of 67 ALS patients and 31 healthy controls who obtained at least one follow-up scan after a median of 6 months, longitudinal FA changes showed significant alterations in ALS patients compared with healthy controls in all ALS-related tracts as well as for the grand average of all tract systems (Kassubek et al., 2018b).

By applying the *in vivo* categorization cascade at the individual level (Kassubek et al., 2014), staging categorization for the baseline scans of 387 ALS patients revealed that 72% of the ALS patients were categorized into ALS stages with a homogeneous distribution over the stages. The longitudinal follow-up study with 67 patients with ALS demonstrated that 27% of the longitudinally scanned ALS patients showed an increase in ALS stage after about 4 months, while the other ALS patients remained stable or had already been classified as ALS stage 4 (Kassubek et al., 2018b).

### The Unbiased Approach Confirms Results at the Cross-Sectional and Longitudinal Group Level

A multicentre study of eight contributing centers with 253 ALS patients and 189 healthy controls (Müller et al., 2016) confirmed the most significant alterations to be localized in the CST (corresponding to stage 1) and found additional significant WM tract changes in the frontal lobe, the brainstem, and hippocampal regions (corresponding to stages 2–4). The localization of these DTI-based *in vivo* results were in accordance with the definition of the post-mortem neuropathological stages (Brettschneider et al., 2013; Braak et al., 2017).

In a longitudinal study with 67 ALS patients and 31 healthy controls and an average inter-scan interval of 6 months (Kassubek et al., 2018b), longitudinal significant FA alterations were also observed in the CST, the frontal lobe, the brainstem, and in hippocampal regions, that way imaging longitudinal alterations of FA during disease progression.

### Hypothetical Longitudinal FA Dependence in ALS Patients

**FIGURE 1:**
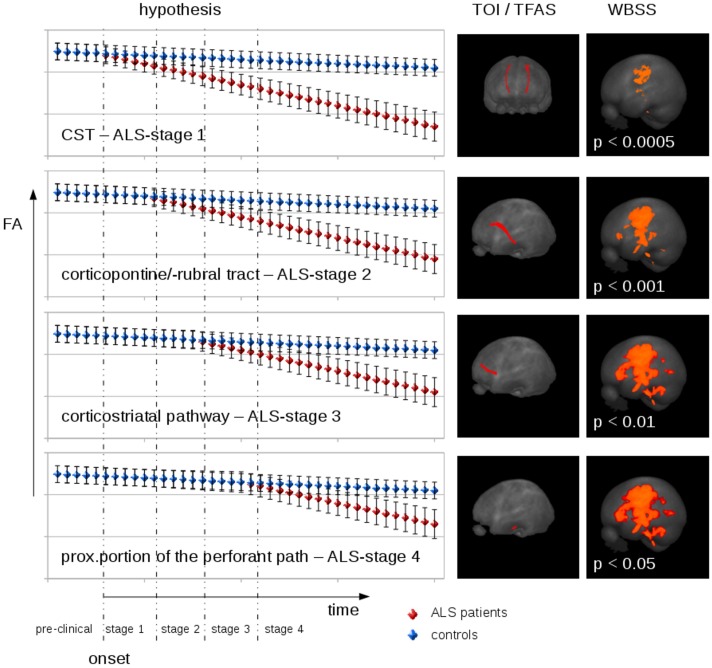
Hypothetical FA development/decrease model for the mean FA values in ALS staging related tract systems. Left panel: At baseline, mean FA was supposed to be identical in patients and controls (with individual error bars). After a certain time interval after disease onset, FA alterations appear first in the CST (related to ALS stage 1). During the disease course, these FA alterations manifest, and FA alterations in frontal and prefrontal areas as well as in the brain stem are observed (corticopontine and corticorubral tract as well as corticostriatal pathway, related to ALS stages 2 and 3, respectively). With higher disease duration, FA alterations in the CST further decrease and alterations in hippocampal areas (proximal portion of the perforant path, related to ALS stage 4) contributed to the FA alteration pattern. Central panel: Projectional views of fiber tracts used for tractwise fractional anisotropy statistics (TFAS) for each of the four stages. Right panel: Projectional views of the corresponding whole brain-based spatial statistics (WBSS).

The cross-sectional and longitudinal FA alterations in ALS patients for unbiased WBSS and hypothesis-guided TFAS suggested a hypothetical FA alteration model for the mean FA values in ALS staging-related tract systems (**Figure 1**). After a certain time interval after disease onset in the ALS patients, FA alterations at the group level appear first in the CST; these FA alterations increase during the disease course, and FA alterations in the corticopontine and corticorubral tract as well as in the corticostriatal pathway can be observed. Finally, FA alterations in the proximal portion of the perforant path contribute to the FA alteration pattern. This hypothetical course is based upon the assumption of almost linear FA alterations. However, there is no proof yet which mathematical model (linear or polynomial) could be assumed for the FA decrease. A solution to this challenge could be the analysis of high-frequency DTI scanning (monthly or even bi-weekly) in a group of about 10 ALS patients during the course of the disease.

A study with 65 DTI scans from ALS patients and healthy controls with several follow-up measurements (Baldaranov et al., 2017) showed an FA decrease in the CST that correlated with the revised ALS functional rating scale (ALS-FRS-R – Cedarbaum et al., 1999). In other studies, both the clinical severity as assessed by the slope of the ALS-FRS-R and the disease duration significantly correlated with the resulting staging scheme (Kassubek et al., 2014, 2018b). Furthermore, the results were recently supplemented by neuropsychological data: 139 patients with ALS were tested with the Edinburgh Cognitive and Behavioral ALS screen (ECAS), in addition to DTI brain measures of pathological spread. Executive function, memory and disinhibited behavior were selected for cognitive staging criteria, since these cognitive functions are attributed to cerebral areas analogous to the pattern of MRI markers of TDP-43 pathology, showing that cognitive impairment follows specific patterns in ALS and, in analogy to DTI-based staging, these patterns are useful to set up a cognitive staging (Lulé et al., 2018).

## Application of the *In Vivo* Staging Approach to Phenotypic Variants of Als

### Lower Motor Neuron Disease and Primary Lateral Sclerosis

The current revision of the El Escorial criteria for ALS addressed restricted phenotypes in the sense of clinical variants (Ludolph et al., 2015). Adult LMND without clinically overt upper motor neuron (UMN) pathology accounts for about 10% of all cases of MND types and is also traditionally named progressive muscular atrophy (PMA) (Norris et al., 1993; Traynor et al., 2000). On the other hand, PLS is considered a MND which almost exclusively affects UMN (Wais et al., 2017).

In a monocentric study of 37 LMND patients vs. 53 healthy controls, WM microstructure showed characteristic alteration patterns in patients with LMND (clinically differentiated in fast and slow progressors according to van den Berg-Vos et al., 2003), especially along the CST with regional FA reductions in the motor system; the TOI-based tract-specific analysis in fast progressing LMND showed significant FA reductions in ALS-related tracts beyond the CST when compared to slow progressors or healthy controls (Rosenbohm et al., 2016). These results were confirmed by a bicentric study of 65 LMND patients compared to 92 matched healthy controls and 101 matched ALS patients with a “classical” phenotype: the tract-specific analysis demonstrated significant alterations in ALS-related tract systems for fast progressing LMND patients vs. slow progressors and healthy controls (Müller et al., 2018a).

There is also a longstanding debate if PLS could be classified as a disease entity separate from ALS or as a slowly progressing ALS variant with UMN predominance (Singer et al., 2007). In the revision of the El Escorial criteria, PLS is described as a restricted phenotype that evolves into ALS in the majority of patients (Ludolph et al., 2015). *In vivo*, the analysis of WM integrity by regional FA reductions in 50 PLS patients vs. 50 controls showed the alterations along the CST and additionally in frontal and prefrontal brain areas in PLS and ALS patients (Müller et al., 2018b). The ALS-staging-related tract-specific analysis demonstrated identical alterations of ALS-related tract systems for PLS and ALS when compared with controls and showed no differences for the comparison between ALS and PLS (Müller et al., 2018b).

## Application of the *In Vivo* Staging Approach to Behavioral Variant of Frontotemporal Dementia

The characteristic longitudinal distribution pattern of the underlying pTDP-43 pathology in the behavioral variant of frontotemporal dementia (bvFTD) across specific brain regions was demonstrated (Brettschneider et al., 2014). The *in vivo* staging approach was transferred to bvFTD (without MND) and showed an alteration pattern in the involved major WM tracts (Kassubek et al., 2018a): the TOIs of bvFTD-pattern 1 (uncinate fascicle), 2 (corticostriatal pathway) and 4 (optic radiation) demonstrated significant differences for bvFTD patients vs. controls, whereas the TOI representing the CST (bvFTD-pattern 3) showed no differences for bvFTD vs. controls. Aspects of the heterogeneous neuropathology of bvFTD which is based upon pTDP-43 only in about 50% of the cases are an issue of discussion (Kassubek et al., 2018a).

## Discussion

In this review, the approach to use DTI metrics in the assessment of axonal damage and myelin degradation in ALS is specifically addressed. An unbiased voxelwise comparison by WBSS (Müller et al., 2012) is an approach to assess microstructural alterations with an imaging resolution in the order of millimeters. WBSS directly compares DTI metrics of subjects at the group level after stereotaxic normalization for the whole brain without any prior restriction to specific brain areas. On the other hand, a tractwise comparison by TOI-based TFAS (Müller et al., 2007b) addresses DTI-based alterations along specific tract structures both at the group level and at the individual level; the hypothesis-guided TOI approach provides a higher statistical accuracy compared to voxelwise analysis since the whole tract structure is taken into account. An alternative approach to assess ALS-related microstructural alterations is TBSS (Smith et al., 2006; Agosta et al., 2010) that aims at analyzing changes in WM across individuals, that way relying on the precise changes in WM across individuals. TBSS is a probabilistic method that generates multiple solutions to reflect the variability or uncertainty of the estimated fiber orientation restricting the statistical comparisons to the centers of WM tracts after non-linear registration (using FA measurements to realign subjects and extract the centers of WM tracts).

### *In vivo* Imaging of TDP-43 Pathology in ALS and Its Variants

Post-mortem studies demonstrated a concept for patterns of TDP-43 pathology in ALS with a sequential progression of pTDP-43 aggregates (Braak et al., 2013), the task remained to investigate if *in vivo* neuroimaging measures might be identified that were consistent with these patterns of pTDP-43 progression (Kassubek et al., 2018a). The TOI-based staging approach (Kassubek et al., 2014) was able to map *in vivo* the proposed neuropathological progression of ALS cross-sectionally as well as longitudinally, that way supporting DTI as a candidate technical marker to image ALS stages *in vivo* (Kassubek et al., 2018b). The microstructural alterations were supplemented by alterations in functional brain organization: specific intrinsic functional connectivity networks revealed significantly increased functional connectivity for the motor network (as the correlate of the neuropathological stage 1), the brainstem network (neuropathological stage 2), the ventral attention network (neuropathological stage 3), and the default mode/hippocampal network (neuropathological stage 4) in a cross-sectional as well as in a longitudinal study design (Schulthess et al., 2016). Increased functional connectivity is strongly indicative for abnormal brain functioning. First, patterns of increased functional connectivity in ALS that result from abnormally strong functional coupling within a specific functional brain network have been attributed to a gradual loss of the inhibitory influence (Douaud et al., 2011). Second, the patterns of increased functional connectivity also present as a network expansion (Schulthess et al., 2016) which is a commonly observed phenomenon in neurodegenerative diseases (Gorges et al., 2015). A straightforward interpretation of adaptive changes is that additional brain areas become functionally integrated, i.e., additional functionally segregated resources are recruited for compensating the ongoing cell loss in within-network modules in order to maintain “normal” performance (Hillary and Grafman, 2017). The application of the *in vivo* techniques to specific MND phenotypes (ALS variants) demonstrates central nervous system involvement of the corticofugal tracts in fast progressive LMND, in support of the hypothesis that LMND is an ALS variant (Müller et al., 2018a). Furthermore, the clinical approach to the phenotype of PLS as an ALS variant was confirmed, in accordance with the latest revision of the El Escorial criteria (Agosta et al., 2015a; Ludolph et al., 2015), in favor of the conclusion that these patients can be treated like ALS and also may be included into clinical trials of ALS (Müller et al., 2018a).

### Hypothesis Guided Tract-Based Analysis

The DTI-based TOI approach is a microstructural correlate of the progressive pathological process; this analysis technique identifies defined anatomical tract systems that represent the proposed progression patterns based upon histopathology (Braak et al., 2013) and are not *per se* determined by a data-driven analysis (Kassubek et al., 2018a). The approach of analyzing a “propagation pattern” is longitudinal in nature. Thus, the analysis according to the progression concept – which has been developed on the basis of cross-sectional post-mortem data – targets the identification of patterns that can be consistently found in a diverse group of neurodegenerative disorders, each of which entails the aggregation of abnormal protein inclusions in characteristic locations (Jucker and Walker, 2013). The longitudinal access of categorizing patients with ALS could be by longitudinal DTI scans followed by confirmation by post-mortem pathology analyses, i.e., the combination of the *in vivo* staging with post-mortem classification in the same subjects. However, the availability of such data is limited. The role of other neuroimaging modalities including molecular imaging has to be evaluated in future studies.

### Limitations

A limitation of the staging categorization is that only about 80% of the MND patients could be categorized. This is a technique-immanent limitation as thresholds for the differentiation between patients and controls were defined in a data-driven approach. Due to an incomplete separation between ALS patients and controls (the sensitivity is about 80%), not all patients would be classifiable (Kassubek et al., 2014). The definition of optimized thresholds by repeated control scans or an increased number of control scans might increase the sensitivity and thus the percentage of categorized MND patients. A further limitation of present neuroimaging approaches is the lack of autopsy-confirmed data (Kassubek et al., 2018a); thus, the TOI-based analysis only provides a plausible surrogate pattern for *in vivo* “staging” for the pathology in the ALS cohorts. Finally, since DTI is a quantitative imaging technique, suboptimal acquisition, data processing and analysis approaches can affect the quality and reliability of DTI-derived metrices (Jones, 2010).

### Summary

Many neurodegenerative diseases feature characteristic patterns of early neuronal and regional vulnerability, with increasing evidence that misfolded protein aggregates can spread by a self-perpetuating process, and novel neuroimaging techniques can help elucidating how these disorders spread across brain networks (Agosta et al., 2015b). Measurement of WM tract involvement seems to be a valid surrogate to assess the *in vivo* spreading of pathological proteins and seems to be a valid approach to provide insights into the trajectory of processes of neurodegeneration (Agosta et al., 2015b) in order to move neuroimaging “from snapshots to motion picture” according to Schuster and co-workers (Schuster et al., 2015).

In ALS as one of the neurodegenerative diseases with such a propagation pattern, the analysis of the neuropathologically defined structures demonstrated a characteristic alteration pattern of the involved WM pathways cross-sectionally as well as longitudinally (Kassubek et al., 2018a); at present, no direct neuroimaging marker for pTDP-43 exists, but previous neuropathological studies have shown the correlation between the degree of pTDP-43 aggregation and axonal loss (Geser et al., 2009). The DTI-based analysis of microstructural integrity is a different approach compared to analysis techniques like regional

volumetric studies that directly measure regional atrophy or intrinsic functional connectivity analysis (Filippi et al., 2015). Thus, the investigation of microstructural integrity by the DTI/TOI-based approach has potential to serve as a non-invasive *in vivo* neuroimaging marker.

The DTI-based techniques have the potential for future use in the work-up of individual patients, they potentially enlarge the spectrum of non-invasive biological markers as a neuroimaging-based read-out for clinical studies (Kassubek et al., 2018a). These studies also could be used for the identification of patients that could be elected for trials targeting at treating the specific histopathologic abnormalities causing MND (Kassubek et al., 2018a). DTI-based scores may provide a different target information to currently available scores for longitudinal screening, as a candidate read-out for future disease-modifying strategies on the transmission of TDP-43 in ALS (Kassubek et al., 2018b).

## Author Contributions

H-PM and JK conceived and designed the study, collected and interpreted the data, and wrote the manuscript.

## Conflict of Interest Statement

The authors declare that the research was conducted in the absence of any commercial or financial relationships that could be construed as a potential conflict of interest.
